# Pre-harvest sprouting resistance and haplotype variation of *ThVp-1* gene in the collection of wheat-wheatgrass hybrids

**DOI:** 10.1371/journal.pone.0188049

**Published:** 2017-11-13

**Authors:** A. A. Kocheshkova, P. Yu. Kroupin, M. S. Bazhenov, G. I. Karlov, A. A. Pochtovyy, V. P. Upelniek, V. I. Belov, M. G. Divashuk

**Affiliations:** 1 Center for Molecular Biotechnology, Russian State Agrarian University–Moscow Timiryazev Agricultural Academy, Moscow, Russia; 2 All-Russia Research Institute of Agricultural Biotechnology, Moscow, Russia; 3 Department of Distant Hybridization, N. V. Tsitsin Main Botanical Garden of Russian Academy of Sciences, Moscow, Russia; McGill University, CANADA

## Abstract

The germplasm collection of 87 wheat-wheatgrass hybrids developed in Tsitisin Main Botanical Garden (Russia, Moscow) was evaluated for resistance to pre-harvest sprouting (PHS) by spike sprouting (SS) and germination index (GI) assays as well as for spike and grain features. The PHS resistance variation and haplotype polymorphism of the wheatgrass *ThVp-1* and wheat *TaVp-1B* genes orthologues of *Vp-1* was revealed in the studied collection. Four haplotypes of *ThVp-1* were revealed: *ThVp-1a* (41% of the entries), *ThVp-1b* (13%), *ThVp-1c* (29%), and *ThVp-1d* (15%). The association between the allelic state of *ThVp-1* and PHS resistance in the wheat-wheatgrass hybrids was shown: haplotype *ThVp-1d* of the wheatgrass *Vp-1* gene is significantly associated with reduced PHS in the wheat-wheatgrass hybrids (mean SS 0.33, mean GI 0.64). The resistant entries may be perspective as a source of PHS resistance in the development of commercial cultivars of perennial wheat.

## Introduction

Wheat-wheatgrass hybrids are partial amphidiploids produced by crossing *Trtitcum* species and various wheatgrass species. Bread wheat (*T*. *aestivum* L., 2n = 6x = 42, genome composition BBAADD) and intermediate wheatgrass (*Thinopyrum intermedium* (Host) Barkworth & D.R. Dewey, 2n = 6x = 42, genome composition J^vs^J^vs^J^r^J^r^StSt) or tall wheatgrass (*Th*. *ponticum* (Podp.) Z.-W. Liu & R.-C. Wang, 2n = 10x = 70, genome composition JJJJJJJ^s^J^s^J^s^J^s^) are the most commonly used for the development of wheat-wheatgrass hybrids. The genetically stable wheat-wheatgrass hybrids are mainly octaploid (2n = 56), carrying 42 chromosomes of wheat and 14 chromosomes from various subgenomes of wheatgrass. Different wheat-wheatgrass amphidiploids carry various combinations of wheatgrass chromosomes [1–5].

The wheat-wheatgrass hybrids were developed in many countries, including Russia, USA, Germany, Canada and China. N. V. Tsitsin was the first who crossed wheat and wheatgrass (*Thinopyrum* sp.) in order to obtain perennial wheat in the 1920s and whose cultivars of partial amphiploid wheat-wheatgrass hybrids were recommended for cultivation at an industrial scale in the former USSR in the 1970s [6]. The aim of the development of wheat-wheatgrass hybrids was to combine the quality of bread wheat with economically beneficial properties of wheatgrass, such as perennial life, winter hardiness and frost resistance, resistance to diseases and pests, and tolerance to abiotic stresses (cold, drought, salinity, etc.). Despite the great potential as a grain or forage crop, wheat-wheatgrass hybrids are currently not cultivated at an industrial scale. However, recently, wheat-wheatgrass hybrid once again attracted attention as a perspective crop [4, 5, 7, 8]. Perennial wheat is an example of a transformative technology, having a number of technological and economic advantages over annual crops: perennial wheat is able to reduce the impact on the environment, is less expensive to manage and can provide stable yields on marginal lands [9, 10]. Wheat-wheatgrass hybrids differ in resistance to diseases and pests [11, 12]. Several resistance genes to fungal, viral diseases and pests were transferred from the genomes of intermediate and tall wheatgrasses into wheat genome [13, 14]. Thus, wheat-wheatgrass hybrids can be used as a breeding bridge between wheat and wheatgrass as a source of valuable traits genes for wheat improvement.

Pre-harvest sprouting (PHS) poses a serious problem for production of cereals including wheat in many grain-producing regions of Russia, Canada, USA and China and needs improvement by breeding [15–17]. PHS occurs in response to heavy rains and dew during harvest and can lead to serious yield losses and reduced grain quality. Flour produced from sprouted grain has a low falling number because of high activity of alpha-amylases that breaks down starch and reduces baking quality [18, 19].

The germination of grain is influenced by a large number of environmental and inherited genetic factors, among the latter the most important are spike morphology and seed dormancy. PHS resistance is influenced by such inherited spike features as spike shape, presence of awns, openness of florets, glume rigidity and germination inhibitors in husks [20], epicuticular wax of glumes, glume adherence, spike inclination and others [21]. Seed dormancy is determined by seed coat and embryo features [22]. Seed coat determines the rate of moisture penetration into the seed. In addition, the seed coat may contain germination inhibitors. The red color of wheat grain is associated with dormancy provided by seed coat and determined by gene *R* mapped to distal region of homeological group 3 [16, 22, 23–28]. The effect of the red color of seed coat may be explained by a genetic linkage between PHS resistance genes and the *R* gene or it may be due to a pleiotropic effect of the *R* gene [23]. Later it was shown that the *R* genes for grain color are transcriptional activators of the flavonoid synthesis genes *TaMyb10* [27].

A total of 110 quantitative trait loci (QTLs) or loci associated with PHS resistance in wheat have been mapped to all wheat chromosomes [29–32], the majority of them are located on the group three chromosomes [33–38] and chromosome 4A [30, 39–41]. Consequently, genes underlying these QTLs were identified such as *TaPHS1* for *Qphs*.*pseru-3AS* on 3AS [42] and most likely *TaMKK3-A* for *Phs-A1* on 4AL [43]. There is increasing evidence that epigenetic changes through DNA and histone methylation may play a role in seed dormancy and, therefore, may determine the PHS resistance of cultivars [44, 45]. In cereals, the role of *ARGONAUTE4_9* class genes in wheat and barley PHS resistance was explored recently and the correlation between DNA methylation status, polymorphism in *AGO802B* and PHS resistance was demonstrated in wheat [46, 47].

The *Viviparous-1* (*Vp-1*) gene is an important regulator of late embryogenesis in maize and a regulator of late embryo development in bread wheat [48]. *Vp-1* plays an important role in processes of seed maturation, such as dehydration and the transition to dormancy in various plant species [49–52]. The *Vp-1* gene encodes an important dormancy-related transcription factor that is involved in the abscisic acid (ABA) signal transduction: a positive correlation was observed between seed dormancy and embryo sensitivity to ABA [53, 54].

The *Vp-1* genes of wheat, *TaVp-1*, are localized on the long arms of the homeologous group 3 chromosomes. Six alleles of *TaVp-1B* were identified and designated as *TaVp-1Ba*, *TaVp-1Bb*, *TaVp-1Bc*, *TaVp-1Bd*, *TaVp-1Be*, and *TaVp-1Bf* [28, 55–57]. The alleles *TaVp-1Bb* and *TaVp-1Bc* detected by an STS molecular marker are associated with higher seed dormancy and PHS resistance [28]. For the *TaVp-1A* gene, also some allelic variations associated with PHS resistance were detected [58]. In general, allelic variants of *Vp-1* affect PHS resistance in white-grained wheat. However, the effect of the allelic state of the *Vp-1* gene on PHS resistance was shown in red-grained triticale depending on the intensity of the grain color [59].

At the present time, two molecular markers based on the sequences of the orthologous *Vp-1* genes of *Thinopyrum intermedium*, *Th*. *ponticum*, *Th*. *bessarabicum* (Savul. & Rayss) and *Pseudoroegneria spicata* (Pursh) have been developed. These markers allow for the identification of various alleles of the *Vp-1* gene orthologue of wheatgrass (*ThVp-1*) in a wheat background [60, 61]. The primers for the markers were designed on the conservative regions while the sequence between them is highly polymorphic and can be distinguished directly by PCR using the STS marker Vivip [61] or with preliminary restriction endonuclease digestion using the CAPS marker Vp1BB4_*Hae*III [60]. The effect of *ThVp-1* on seed dormancy has never been explored.

Although PHS resistance is important for both the development of commercial cultivars of wheat-wheatgrass hybrids and for wheat breeding using wheat-wheatgrass germplasm, this trait has not been studied yet in wheat-wheatgrass hybrids. The aim of this work is to study the germplasm collection of the wheat-wheatgrass hybrids for PHS resistance using different assays, and to assess the effect of grain color, spike parameters and *Vp-1* of wheat and wheatgrass on PHS resistance.

## Materials and methods

### Plant materials

The wheat-wheatgrass hybrid germplasm collection includes 87 entries bred by N.V. Tsitsin, V.F. Lyubimova, V.I. Belov et al. (Department of Distant Hybridization, Tsitsin Main Botanical Garden, Russian Academy of Sciences) (Table 1). Bread wheat cv. Nota and *Th*. *intermedium* accession PI 401200 (Germplasm Research International Network) were used as controls for the *TaVp-1* and *ThVp-1* genes, respectively, in PCR experiments.

**Table 1:** Characteristics of the wheat-wheatgrass hybrid collection.

Entry	Wheatgrass *ThVp-1* haplotype	Wheat *Vp-1* allele	GI	SS*	Grain color	Glume color	Awn presence (+) / absence (–)	Threshability
12	*ThVp-1d*	*Vp-1Ba*	0.69	0.56	light red	white	–	easy
33	*ThVp-1c*	*Vp-1Ba*	0.94	0.48	light red	white	+	easy
49	*ThVp-1a*	*Vp-1Ba*	0.81	0.51	light red	white	–	easy
90	*ThVp-1b*	*Vp-1Bc*	0.74	0.58	light red	white	+	easy
166	*ThVp-1a*	*Vp-1Ba*	0.71	0.33	dark red	white	–	easy
168	*ThVp-1b*	*Vp-1Bc*	0.62	0.51	dark red	red	–	easy
186	*ThVp-1a*	*Vp-1Ba*	0.85	0.52	light red	white	–	easy
192	*ThVp-1a*	*Vp-1Ba*	0.91	0.55	light red	white	–	easy
237	*ThVp-1c*	*Vp-1Ba*	0.89	0.5	light red	white	+	hard
243	*ThVp-1c*	*Vp-1Ba*	0.68	0.45	light red	white	–	easy
249	*ThVp-1c*	*Vp-1Ba*	0.73	0.61	dark red	white	+	easy
548	*ThVp-1d*	*Vp-1Ba*	0.75	0.66	light red	red	–	easy
1375	*ThVp-1d*	hg	0.78	0.42	dark red	white	–	hard
1382	*ThVp-1a*	*Vp-1Ba*	0.86	0.36	light red	white	–	easy
1451	*ThVp-1c*	*Vp-1Bc*	0.87	0.01	blue	white	–	hard
1512	*ThVp-1b*	*Vp-1Bc*	0.64	0.49	light red	red	–	easy
1514	*ThVp-1d*	*Vp-1Bc*	0.95	0.5	dark red	red	–	easy
1533	*ThVp-1a*	*Vp-1Ba*	0.54	0.04	dark red	white	–	easy
1546	*ThVp-1c*	hg	0.77	0.54	light red	white	–	easy
1626	*ThVp-1d*	*Vp-1Ba*	0.76	0.41	light red	red	–	easy
1646	*ThVp-1a*	*Vp-1Ba*	0.90	0.40	light red	white	–	easy
1654	*ThVp-1c*	*Vp-1Bc*	0.62	0.01	blue	white	–	hard
1674	*ThVp-1b*	*Vp-1Bc*	0.78	0.44	light red	white	+	easy
1689	*ThVp-1a*	*Vp-1Bc*	0.73	0.44	light red	white	–	easy
1690	*ThVp-1a*	*Vp-1Ba*	0.78	0.66	light red	white	–	easy
1692	*ThVp-1b*	*Vp-1Bc*	0.60	0.62	dark red	white	–	easy
1735	*ThVp-1d*	*Vp-1Ba*	0.42	0.08	light red	white	–	easy
1737	*ThVp-1a*	hg	0.61	0.48	dark red	white	–	easy
1744	*ThVp-1a*	*Vp-1Ba*	0.73	0.12	light red	white	–	easy
1745	*ThVp-1d*	*Vp-1Ba*	0.61	0.09	light red	white	–	easy
1748	*ThVp-1a*	*Vp-1Ba*	0.72	0.07	light red	white	–	easy
1755	*ThVp-1a*	*Vp-1Ba*	0.90	0.45	light red	red	–	easy
1761	*ThVp-1a*	*Vp-1Bc*	0.91	n/a	dark red	white	–	easy
1770	*ThVp-1b*	*Vp-1Ba*	0.99	0.58	light red	white	–	easy
1772	*ThVp-1c*	*Vp-1Ba*	0.94	0.81	light red	white	small awns	easy
1774	*ThVp-1c*	*Vp-1Ba*	0.45	0.12	dark red	white	small awns	easy
1777	*ThVp-1a*	*Vp-1Ba*	0.60	0.29	light red	white	–	easy
1780	*ThVp-1d*	*Vp-1Ba*	0.61	0.01	light red	white	–	easy
1783	*ThVp-1a*	hg	0.71	0.33	light red	white	–	easy
1784	*ThVp-1c*	*Vp-1Bc*	0.95	0.81	light red	white	–	easy
1788	*ThVp-1a*	hg	0.93	n/a	light red	white	–	easy
1792	*ThVp-1c*	hg	0.93	0.89	light red	red	–	easy
1803	*ThVp-1c*	*Vp-1Bc*	0.97	0.86	light red	white	small awns	easy
1805	*ThVp-1c*	*Vp-1Ba*	0.92	0.65	light red	white	–	easy
1842	*ThVp-1a*	*Vp-1Bc*	0.87	n/a	light red	white	–	easy
1866	*ThVp-1a*	*Vp-1Ba*	0.74	0.52	light red	red	–	easy
1868	*ThVp-1c*	hg	0.85	0.46	light red	white	–	easy
1869	*ThVp-1c*	*Vp-1Ba*	0.87	0.29	dark red	white	small awns	easy
1872	*ThVp-1c*	*Vp-1Ba*	0.99	0.69	dark red	red	+	easy
1874	*ThVp-1a*	*Vp-1Bc*	0.86	0.09	dark red	red	–	easy
1876	*ThVp-1d*	hg	0.52	0.25	light red	white	–	easy
1877	*ThVp-1a*	hg	0.81	0.51	light red	white	–	easy
1878	*ThVp-1a*	*Vp-1Ba*	0.68	0.45	light red	white	–	easy
2087	*ThVp-1b*	*Vp-1Bc*	0.73	0.41	dark red	white	–	easy
3215	*ThVp-1c*	hg	0.85	0.44	light red	white	–	easy
3240	*ThVp-1c*	*Vp-1Bc*	0.80	0.54	light red	white	–	easy
4015	*ThVp-1b*	*Vp-1Bc*	0.68	0.64	dark red	white	–	easy
4023	*ThVp-1b*	*Vp-1Bc*	0.80	0.59	dark red	white	–	easy
4044	*ThVp-1a*	*Vp-1Ba*	0.98	0.86	light red	white	–	easy
4056	hg	hg	0.74	0.89	n/a	white	–	easy
4061	*ThVp-1b*	*Vp-1Ba*	0.83	0.64	light red	white	–	easy
4082	*ThVp-1a*	*Vp-1Ba*	0.74	0.89	light red	white	small awns	easy
5156	*ThVp-1d*	*Vp-1Bc*	0.64	0.47	light red	white	–	easy
5542	*ThVp-1a*	hg	0.82	n/a	light red		–	easy
5795	*ThVp-1d*	*Vp-1Ba*	0.77	0.43	light red	white	–	easy
1416-bo	*ThVp-1a*	*Vp-1Bc*	0.42	0.15	dark red	white	–	easy
1416-o	*ThVp-1a*	*Vp-1Bc*	0.84	0.92	light red	white	+	easy
150-b	*ThVp-1a*	*Vp-1Ba*	0.75	0.63	light red	white	small awns	easy
150-k	*ThVp-1c*	*Vp-1Bc*	0.41	0.2	light red	red	small awns	easy
1665-o	*ThVp-1c*	hg	0.75	0.65	light red	white	+	easy
1665-h	*ThVp-1c*	hz	0.67	n/a	light red	white	small awns	easy
1765-b	*ThVp-1d*	*Vp-1Ba*	0.71	n/a	light red	white	–	easy
1765-k	*ThVp-1d*	*Vp-1Ba*	0.15	0.02	light red	red	–	easy
1795-so	*ThVp-1a*	*Vp-1Ba*	0.69	n/a	dark red	white	+	easy
1795-slo	hg	*Vp-1Ba*	0.66	0.47	dark red	white	small awns	easy
1804-b	*ThVp-1a*	hz	0.87	n/a	light red	white	–	easy
1804-k	*ThVp-1a*	*Vp-1Bc*	0.82	0.43	light red	red	–	easy
1807-o	*ThVp-1c*	*Vp-1Ba*	0.81	0.57	light red	white	+	easy
1807-h	*ThVp-1c*	*Vp-1Ba*	0.85	n/a	light red	white	small awns	easy
1861-bo	*ThVp-1a*	hz	0.82	n/a	light red	white	–	easy
1865-bkbk	*ThVp-1c*	*Vp-1Bc*	0.94	n/a	dark red	white	–	easy
1865-bkk	*ThVp-1c*	*Vp-1Bc*	0.97	n/a	dark red	red	–	easy
1870-bo	*ThVp-1a*	*Vp-1Ba*	0.80	n/a	light red	white	–	easy
ZP26	*ThVp-1a*	*Vp-1Ba*	0.86	0.60	light red	white	–	easy
M169	*ThVp-1b*	*Vp-1Bc*	0.70	0.44	light red	white	–	easy
M3202	*ThVp-1a*	*Vp-1Ba*	0.84	0.65	light red	white	–	easy
Otrastayushchaya 38	*ThVp-1a*	*Vp-1Bc*	0.79	0.45	light red	white	small awns	easy

GI—germination index.

SS–spike sprouting.

hz–heterozygote.

hg–heterogeneous entry.

n/a–not available.

*—SS was assessed in 74 out of 87 entries due to the lack of plant material.

### Assay of germination index (GI) and spike sprouting (SS)

The entries were grown in the fields of the Department of Distant Hybridization (Tsitsin Main Botanical Garden, Russian Academy of Sciences) at the Snegiri settlement, Istrinsky district of Moscow region (55°51'32"N 37°1'54"E). Germination index was determined according to Walker-Simmons method [62]. The spikes were harvested at full ripeness and threshed manually. 25 seeds in four replicates were placed crease down on moist filter paper in Petri dishes, which were then placed in lit climatic chamber (12 hours day, 12 hours night) at +20°C. The number of germinated seeds was counted daily and removed after counting during 7 days. The seeds were taken as germinated at radical emergence. Water was added to Petri dishes as needed using a sprayer. The remaining seeds were left to germinate for one month to determine their viability. Seeds that did not sprout within a month were excluded from further calculations. The germination index (GI) was calculated according to the formula:

$$GI=\frac{7n_{1}+6n_{2}+5n_{3}+4n_{4}+\ldots +1n_{7}}{7TG},$$

where *TG* were total grains, *n*_*1*_, *n*_*2*_,… *n*_*7*_ were the numbers of seeds germinated on the first, second, and subsequent days until the seventh day.

Spike sprouting assay (SS) of the wheat-wheatgrass entries was conducted in moist chamber on the shelves wrapped in plastic film. The spikes were harvested at full ripeness, steeped in distilled water for an hour, five spikes per each entry were bound into sheaves and placed on the shelves in an upright position. The sheaves were placed into a chamber and sprayed three times a day for 5 minutes each time. Visual counting of clearly sprouted grains was performed at the third day of sprouting provocation. Then, the spikes were dried and threshed to determine the total number of grains in them. The SS value was calculated as a ratio of sprouted grains to the total number of grains.

In addition, the spike morphology was estimated–presence of awns, glume color, grain color, and threshability.

### DNA extraction and molecular markers

The DNA of the wheat-wheatgrass hybrids was extracted from seedlings using CTAB protocol [63].

The PCR analysis was performed using markers Vp1BB4_*Hae*III and Vivip for the wheatgrass *ThVp-1* genes and Vp-1B3 for the wheat *TaVp-1B* gene.

The primers Vp1BB4 were developed by Yang et al. (2007) [28] (Vp-1BB4F: 5ʹ-CAATGAGCTGCAGGAGGGTGA-3ʹ, Vp-1BB4R: 5ʹ-ATCATCCCTAACTAGGGCTACG-3ʹ) and converted by us into the CAPS marker Vp1BB4_*Hae*III [60]. The conditions for PCR amplification were 94^○^C for 1 min; followed by 35 cycles of 95^○^C for 1 min; 64^○^C for 1 min; 72^○^C for 1 min, with a final extension of 72^○^C for 10 min. The PCR products were digested using the *Hae*III endonuclease for 12 h at 37^○^C.

The STS marker Vivip was designed by Kocheshkova et al. (2014) [61] (VivipF: 5ʹ- GGGTGATTTCATCGTGCTT-3ʹ, VivipR: 5ʹ-TCTCCAACACTTGATTTTGACC-3ʹ). The conditions for PCR amplification were 95°C for 7 min, followed by 35 cycles of 95°C for 1 min, 60°C for 1 min and 72°C for 1 min, with the final extension of 72°C for 10 min.

The primers and conditions for PCR amplification of Vp-1B3 are described in Yang et al. (2007) [28].

The PCR fragments were separated on 2% agarose gel with TBE buffer at 6 V/cm field strength with GeneRuler 100 bp DNA Ladder (Thermo Fisher Scientific) as a size marker, stained with ethidium bromide, and visualized using UV light.

### Statistical analysis

The grouping of the data was carried out using Sturges’ rule [64, 65]. Analysis of variance (ANOVA) was performed in the ‘Statistica 6.0’ program. The differences in GI and SS among genotypes with different PCR profiles were tested using Fisher’s least significant difference (LSD) test as in Chang et al. (2010 a,b) [57, 66].

## Results

PHS resistance is determined by two groups of traits: spike morphology (presence of awns, glume adherence, spike inclination, etc.) and grain characteristics (color, germination inhibitors in seed coat, hormonal status, etc.) [15, 16, 62, 67–71]. The effect of the first group can be reflected by evaluating spike sprouting (SS) in intact spikes in moist chamber, and the second group by germination index (GI) of the threshed grains in Petri dishes.

### Spike sprouting assay (SS)

**Fig 1:**
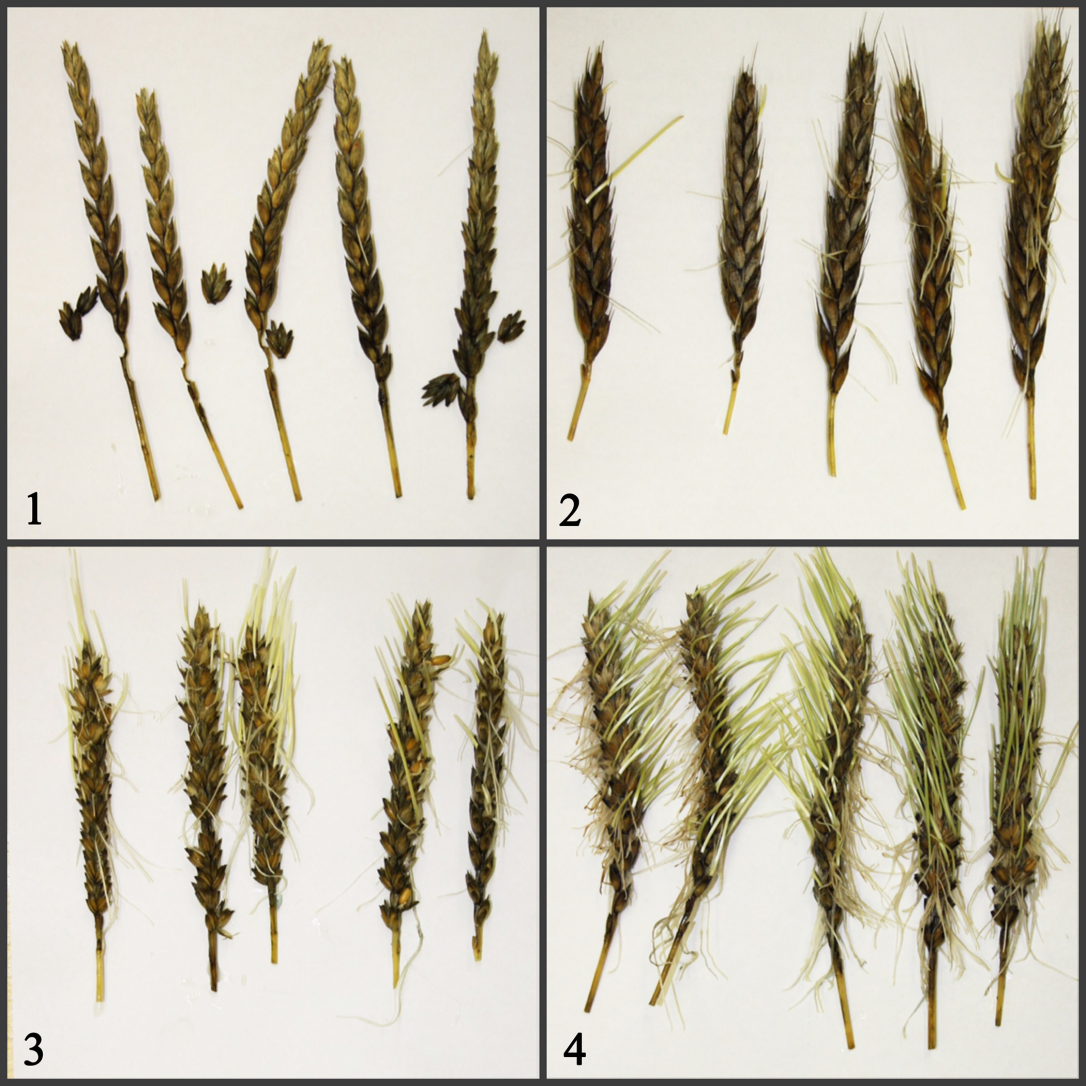
Estimation of the PHS resistance by spike sprouting assay. 1–1451; 2–150-k; 3–1626; 4–4044.

For a set of 74 entries spike sprouting (SS) was estimated (Fig 1, Table 1). The proportion of germinated seeds was determined visually on the third day of spikes wetting.

Using the Sturges’ rule, a set of 74 entries was divided into 8 clusters with the corresponding SS ranges. The most resistant entries sorted to cluster 1 were 1451, 1654, 1780, 1765-k, 1533, 1748, 1735, 1745, 1874, 1744, 1774 (Table 2).

**Table 2:** Classification of the wheat-wheatgrass collection entries based on the results of the SS assay.

Cluster	Range of SS	Number of entries	Wheat-wheatgrass entries
1	0,01–0,12	11	1451, 1654, 1780, 1765-k, 1533, 1748, 1735, 1745, 1874, 1744, 1774
2	0,13–0,24	2	1416-bo, 150-k
3	0,25–0,36	6	1876, 1777, 1869, 166, 1783, 1382
4	0,37–0,48	19	1646, 1626, 2087, 1375, 1804-k, 5795, 1674, 1689, 3215, M169, 1755, 1878, 243, Otrastayushchaya 38, 1868, 1795-slo, 5156, 1737, 33
5	0,49–0,60	17	1512, 1514, 237, 168, 1877, 49, 186, 1866, 1546, 3240, 192, 12, 1807-o, 1770, 90, 4023, ZP26
6	0,61–0,72	11	249, 1692, 150-b, 4015, 4061, 1665-o, 1805, 1690, M3202, 548, 1872
7	0,73–0,84	2	1772, 1784
8	0,85–0,97	6	1803, 4044, 1792, 4056, 4082, 1416-o

### Germination index (GI)

For a set 87 entries the germination index (GI) was evaluated. Using the Sturges’ rule, the set was divided into 8 clusters with the corresponding GI ranges (Table 3).

**Table 3:** Classification of wheat-wheatgrass entries by germination index (GI).

Cluster	Range of GI	Number of entries	Wheat-wheatgrass entries
1	0,15–0,25	1	1765-k
2	0,26–0,35	0	—
3	0,36–0,46	4	150-k, 1416-bo, 1735, 1774
4	0,47–0,56	2	1876, 1533
5	0,57–0,67	11	1777, 1692, 1780, 1745, 1737, 1654, 168, 5156, 1512, 1795-slo, 1665-h
6	0,68–0,78	27	243, 1878, 4015, 12, 1795-so, M169, 166, 1783, 1765-b, 1748, 1744, 2087, 1689, 249, 1866, 90, 4056, 4082, 150-b, 1665-o, 548, 1626, 5795, 1546, 1375, 1674, 1690
7	0,79–0,88	24	Otrastayushchaya 38, 3240, 4023, 1870-bo, 49, 1877, 1807-o, 1804-k, 5542, 1861-bo, 4061, M3202, 1416-o, 3215, 1868, 186, 1807-h, 1874, 1382, ZP26, 1451, 1869, 1842, 1804-b
8	0,89–0,99	18	237, 1646, 1755, 192, 1761, 1805, 1792, 1788, 33, 1772, 1865-bkbk, 1514, 1784, 1803, 1865-bkk, 4044, 1770, 1872

**Fig 2:**
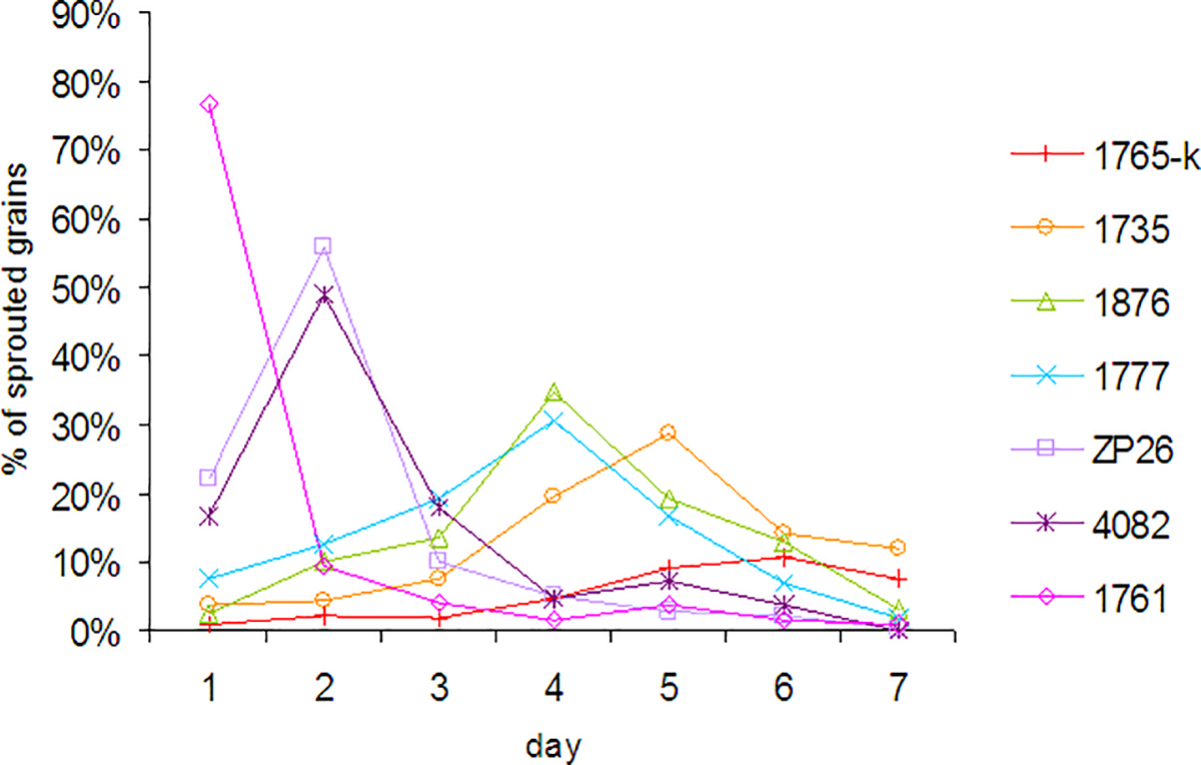
Example of dynamics of grain germination of various wheat-wheatgrass entries in Petri dishes.

The entries of different clusters are characterized by different dynamics of germination (Fig 2). The immediate germination of the majority of grains on the 1st day is represented by 1761 (cluster 8). ZP26 (cluster 7) and 4082 (cluster 6) showed the peak on the 2nd day, 1777 (cluster 5) and 1876 (cluster 4) on the 3rd day, 1735 (cluster 3) on the 5th day. Extended dynamics of germination is intrinsic for 1765-k, the most PHS resistant entry. Therefore, the lower GI value, the more delayed and faint peak of germination can be observed.

Entry 1451 showed both minimum SS value (0.01) and a high GI value (0.87). As 1451 is extremely hard to thresh and has rigid glumes adherent to the caryopsis, its high PHS resistance in the spike is determined primarily by the spike architecture, and the seeds themselves have a low dormancy level. The spike architecture, apparently, also explains PHS resistance in 1748 and 1874 (SS 0.07 and 0.09), which possess low seed dormancy (GI 0.72 and 0.86). 1765-k has low SS (0.02) and low GI (0.15), hence its PHS resistance can be explained equally by the seed and spike characteristics. 1735, 1774 and 150-k showed high resistance in the SS and GI assays, which allows us to consider them as generally resistant to PHS. 4044, 1416-o, 1792, 1803 showed high SS and GI, and thus can be considered as susceptible to PHS. Therefore, the spike features of these entries are unable to provide PHS resistance and to compensate for the low seed dormancy. The entries with low GI and high SS were not revealed (Table 4). That indicates that a high level of seed dormancy is enough for providing acceptable PHS resistance.

**Table 4:** Classification of entries based on the results of germination index (GI) and spike sprouting (SS) assays.

	Low GI	High GI
Low SS	1765-k, 1735, 1744, 150-k.	1451, 1748, 1874
High SS	Not found	4044, 1416-o, 1792, 1803

### Distribution of wheatgrass *ThVp-1* haplotypes

Markers Vp1BB4_*Hae*III and Vivip allow the identification of various alleles of the wheatgrass *ThVp-1* gene in a wheat background [60, 61]. The DNA fragments of Vp1BB4_*Hae*III produced by PCR and subsequent restriction endonuclease digestion were analyzed in all entries. Vp1BB4_*Hae*III enables the identification of additional fragments different from wheat after restriction of the PCR product. Such additional bands were interpreted as fragments amplified from *ThVp-1* [60].

**Fig 3:**
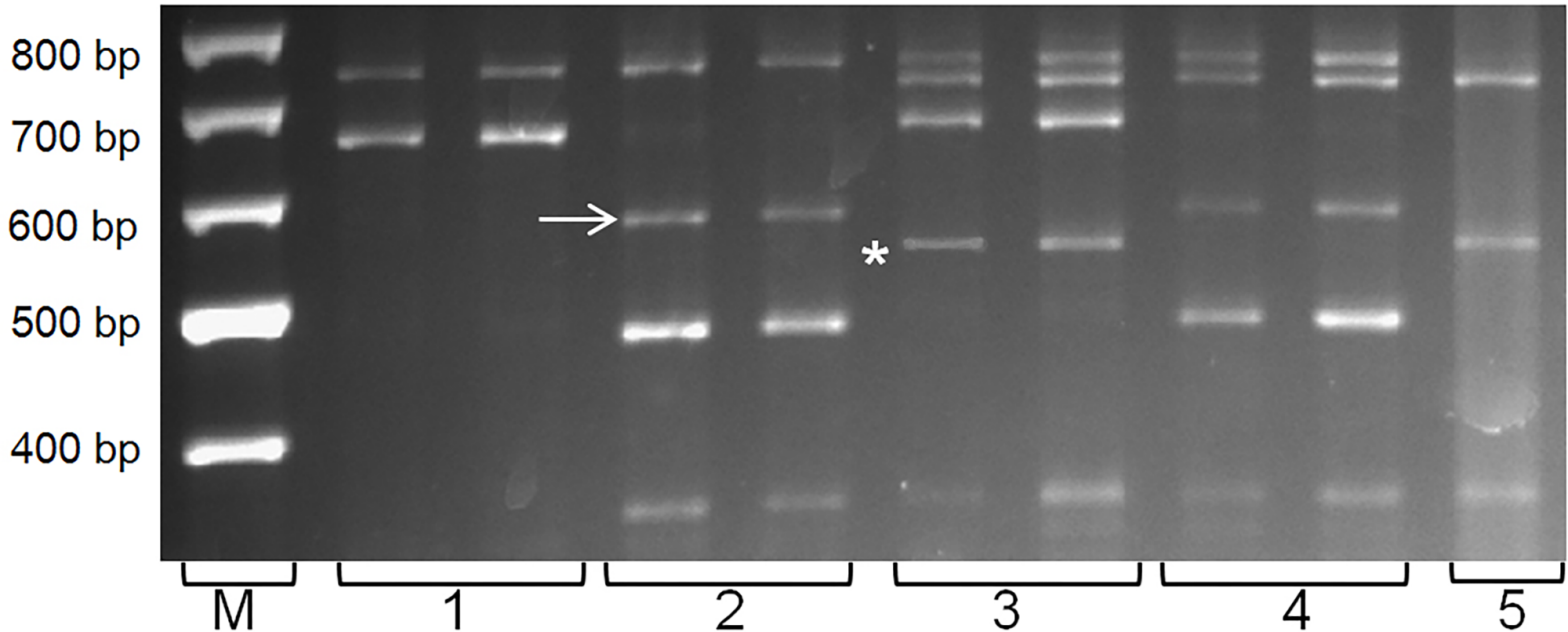
Electrophoresis of the CAPS-marker Vp1BB4_*Hae*III fragments. The ~600 bp (arrow) and ~550 bp (*) are diagnostic fragments for the wheatgrass *ThVp1* gene. M—size standard (100 bp DNA Ladder), 1 –*Triticum aestivum cv*. *Nota*, 2–90, 3–150 k., 4–168, 5 –*Th*. *intermedium* PI 401200.

In our study, the analysis of 87 entries using Vp1BB4_*Hae*III revealed two sizes of additional fragments of *ThVp-1*, namely, approximately 600 bp and approximately 550 bp (Fig 3, Table 5). Consequently, Vp1BB4_*Hae*III can distinguish three possible *ThVp-1* states: presence of the ~600 bp fragment, presence of the ~550 bp fragment and absence of both fragments. No entry with simultaneous presence of both the ~550 and ~600 bp fragments was found.

**Table 5:** The combined results of application of the Vp1BB4_*Hae*III and Vivip markers in the wheat-wheatgrass hybrids.

Wheatgrass *ThVp-1* haplotypes	Marker results		Wheat-wheatgrass entries
	Vp1BB4_*Hae*III	Vivip	
*ThVp-1a*	No specific fragments	No specific fragments	49, 150-b, 166, 186, 192, 1382, 1416-bo, 1416-o, 1533, 1646, 1689, 1690, 1737, 1744, 1748, 1755, 1761, 1777, 1783, 1788, 1795-so, 1804-b, 1804-k, 1842, 1861-bo, 1866, 1870-bo, 1874, 1877, 1878, 4044, 4082, 5542, ZP26, M3202, Otrastayushchaya 38
*ThVp-1b*	~600 bp	No specific fragments	90, 168, 1512, 1674, 1692, 1770, 2087, 4015, 4023, 4061, M169
*ThVp-1c*	~550 bp	~370 bp	33, 150-k, 237, 243, 249, 1451, 1546, 1654, 1665-h, 1665-o, 1772, 1774, 1784, 1792, 1803, 1805, 1807-o, 1807-h, 1865bkbk, 1865-bkk, 1868, 1869, 1872, 3215, 3240
*ThVp-1d*	No specific fragments	~350 bp	12, 548, 1375, 1514, 1626, 1735, 1745, 1765-k, 1765-b, 1780, 1876, 5156, 5795

**Fig 4:**
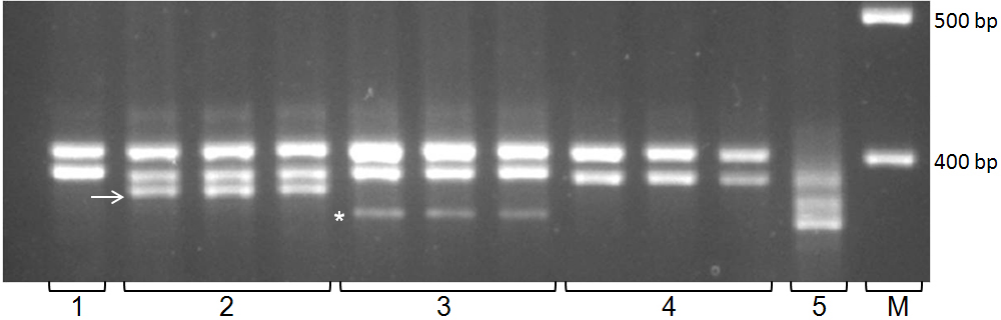
An example of electrophoresis of PCR products of the STS marker Vivip. The DNA fragments indicating the presence of wheatgrass *ThVp-1* are ~350 bp and ~370 bp. 1 –*Triticum aestivum cv*. *Nota*; 2–1665, 3–5156, 4 –ZP26, 5 –*Th*. *intermedium* PI 401200, M–size standard, 100 bp DNA Ladder.

The Vivip marker was designed based on sequences of another polymorphic regions of the orthologous *Vp-1* genes of *Th*. *intermedium*, *Th*. *ponticum*, *Th*. *bessarabicum*, and *P*. *spicata*. The results of the identification of the *ThVp-1* polymorphism using Vivip are independent from Vp1BB4_*Hae*III [61]. In our study, the PCR analysis of 87 entries using Vivip allowed the identification of two additional amplified DNA fragments of wheatgrass origin, namely, approximately 350 bp, and approximately 370 bp (Fig 4). In the studied collection of the wheat-wheatgrass hybrids three possible states of Vivip were revealed: absence of both fragments, presence of the ~350 bp fragment, and presence of the ~370 bp fragment (Fig 4, Table 5).

The analysis of the results of two molecular markers have revealed that the 370 bp fragment detected by Vivip occurs in the same wheat-wheatgrass entries as the 550 bp fragment, which is detected by Vp1BB4_*Hae*III. Thus, fragments ~550 bp of Vp1BB4_*Hae*III and ~370 bp of Vivip detect the same allele of the wheatgrass *ThVp-1* gene.

1795-slo and 4056 showed heterogeneity for the allelic state of *ThVp-1*, so, they were excluded from the further statistical analysis.

The analysis of 87 entries using both markers Vp1BB4_*Hae*III and Vivip showed four possible haplotypes of the wheatgrass *ThVp-1* gene that are present in the studied wheat-wheatgrass hybrids (Table 5). These four variants are characterized by the molecular markers, as follows: 1) Absence of the specific wheatgrass DNA fragments of both markers (designated *ThVp-1a*, 36 entries); 2) Presence of the ~600 bp fragment detected by Vp1BB4_*Hae*III and absence of specific fragments of Vivip (desisgnated *ThVp-1b*, 11 entries); 3) Presence of the ~550 bp fragment of Vp1BB4_*Hae*III and presence of the ~370 bp fragment Vivip (desisgnated *ThVp-1c*, 25 entries); 4) Absence of specific fragments for Vp1BB4_*Hae*III and presence of the ~350 bp fragment revealed by Vivip (desisgnated *ThVp-1d*, 13 entries).

Thus, we have identified a polymorphism of the wheatgrass *ThVp-1* gene in the wheat-wheatgrass hybrid collection and they were grouped into 4 classes (haplotypes) depending on the results of the combined use of Vp1BB4_*Hae*III and Vivip. The distribution of haplotypes in the collection is as follows: the predominant haplotype is *ThVp-1a* (41%), second place is allele *ThVp-1c* (29%), followed by haplotypes *ThVp-1d* (15%) and *ThVp-1b* (13%).

The association between *ThVp-1* haplotypes and PHS resistance based on GI in the entire germplasm collection was evaluated statistically using the analysis of variance (ANOVA) (Table 6).

**Table 6:** Association between *ThVp-1* haplotypes and GI values in the wheat-wheatgrass hybrid collection.

*ThVp-1* haplotype	Number of entries	Mean GI*	Standard deviation	Range
*ThVp-1a*	36	0.78^b^	0.12	0.42–0.98
*ThVp-1b*	11	0.74^ab^	0.11	0.60–0.99
*ThVp-1c*	25	0.82^b^	0.15	0.41–0.99
*ThVp-1d*	13	0.64^a^	0.20	0.15–0.95

* F_observed_ = 4.635 > F_critical_ = 2.717; p = 0.0048.

Different letters in this column indicate significant differences (P<0.05; Fisher’s LSD test) among different *ThVp-1* haplotypes

The ANOVA indicated significant GI differences between the groups of wheat-wheatgrass hybrids with various *ThVp-1* haplotypes. Statistically significant differences were observed between haplotypes ‘a’ and ‘d’ and between haplotypes ‘c’ and ‘d’. Thus, entries with the *ThVp-1d* haplotype had significantly lower average GI than *ThVp-1a* and *ThVp-1c* genotypes confirming the association of *ThVp-1* and seed dormancy.

The ANOVA conducted after arcsin√x transformation of the SS values and Fisher’s F-test revealed no significant differences between the groups of wheat-wheatgrass hybrids with different *ThVp-1* haplotypes. However, LSD test showed that the group of wheat-wheatgrass hybrids with *ThVp-1d* haplotype gave lower value of SS than groups with haplotypes ‘b’ and ‘c’ (Table 7).

**Table 7:** Association between *ThVp-1* haplotypes and SS values in the wheat-wheatgrass hybrid collection.

*ThVp-1* haplotype	Number of entries	Mean SS arcsin√x transformed*	Mean SS reverse transformed	Standard deviation (for arcsin√x transformed means)	Range (non- transformed)
*ThVp-1a*	28	0.73^ab^	0.45	0.27	0.04–0.92
*ThVp-1b*	11	0.83^b^	0.54	0.09	0.41–0.64
*ThVp-1c*	21	0.77^b^	0.49	0.31	0.01–0.89
*ThVp-1d*	12	0.57^a^	0.29	0.28	0.01–0.66

* F_observed_ = 2.13 < F_critical_ = 2.74; p = 0.104.

Different letters in this column indicate significant differences (P<0.05; Fisher’s LSD test) among different *ThVp-1* haplotypes

### Distribution of wheat *TaVp-1* haplotypes

As wheat-wheatgrass hybrids combine wheat and wheatgrass genomes, our collection was also evaluated for the allelic state of the wheat *TaVp-1* gene using the STS marker developed by Yang et al. (2007) [28] (Table 1). The structure of the studied germplasm collection was as follows: 51% of the entries carried allele *TaVp-1Ba*, 31% carried allele *TaVp-1Bc*, 15% showed heterogeneity, 3% were heterozygotes (the latter two groups were not involved in the statistical analysis).

The GI differences between groups of wheat-wheatgrass hybrids with different allelic state of wheat *TaVp-1B* were not significant with similar mean GI values (Table 8).

**Table 8:** Association between *TaVp-1B* alleles (of wheat origin) and GI values in the wheat-wheatgrass hybrid collection.

Allele of wheat *Vp-1B*	Number of entries	Mean GI*	Standard deviation	Range
*Vp-1Bc*	27	0.76	0.15	0.41–0.97
*Vp-1Ba*	44	0.76	0.16	0.15–0.99

* F_observed_ = 0.003 < F_critical_ = 3,982; p = 0.959

No significant differences of SS between groups of wheat-wheatgrass hybrids with different *TaVp-1B* alleles were revealed (Table 9).

**Table 9:** Association between *TaVp-1B* alleles (of wheat origin) and SS values in the wheat-wheatgrass hybrid collection.

Allele of wheat *Vp-1B*	Number of entries	Mean SS arcsin√x transformed	Mean SS reverse transformed*	Standard deviation (for arcsin√x transformed means)	Range (non- transformed)
*Vp-1Bc*	23	0.73	0.46	0.30	0.01–0.92
*Vp-1Ba*	40	0.72	0.45	0.27	0.01–0.89

* F_observed_ = 0.21376 < F_critical_ = 1.74; p = 0.64519

In addition, no statistically significant association was revealed between PHS resistance measured by SS and GI and the presence of awns, glumes color, and grain color (data not shown).

## Discussion

In our work for the first time, the PHS resistance polymorphism in wheat-wheatgrass hybrids was revealed by GI and SS evaluation. In our study, the wheat-wheatgrass hybrids significantly differ from each other in PHS resistance. Similarly, wheat-wheatgrass hybrids are also highly polymorphic in other traits such as diseases resistance, grain yield, post-harvest regrowth ability, forage biomass [4, 5, 72–74]. Among the resistant entries with low GI we identified two types of germination dynamics, namely, an extended germination and delayed peak. In general, the majority of the entries were susceptible to PHS (92% are susceptible to PHS, classes 5–8, Table 3). It may be a serious problem for the introduction of wheat-wheatgrass hybrids as a cereal crop in regions where climate favors PHS. Currently, the main attempts to develop perennial wheat-wheatgrass hybrids as a crop are being made in Australia [4, 5, 72], where PHS is not a relevant problem and probably that is why it receives little attention.

PHS resistance in grasses is controlled by multiple factors. QTLs for PHS resistance have been found on all chromosomes of wheat in different studies [23, 29, 34–36, 75–90]. Many studies of the PHS resistance in wheat is focused on *TaVp-1*, the polymorphism of its haplotypes and its effect on PHS [28, 57–59, 66, 91–94]. In the majority of them the influence of different alleles of *TaVp-1B* and *TaVp-1A* was shown primarily in white-grained wheat, though in some studies the effect of *TaVp-1* was found in red-grained wheat and triticale as well [59, 93]. Given the fact that all wheat-wheatgrass entries possess red or blue grain, which is usually associated with resistance, the absence of resistance in the majority of entries is apparently a result of the negative impact of the wheatgrass genome. For example, a negative effect of chromosome 6Ag^i^ of intermediate wheatgrass on PHS resistance was revealed in the studies of the recombinant inbred substitution lines of wheat [21]. This should be considered in breeding and development of cultivation technology of commercial wheat-wheatgrass cultivars. In addition, one should note, that the proportion of entries demonstrating resistance to sprouting in intact spikes is higher than the proportion of entries demonstrating seed dormancy in the evaluation of GI. Apparently, this is due to the spike architecture of wheat-wheatgrass hybrids. Spikes of wheat-wheatgrass hybrids are generally more dense and hard to thresh in comparison to bread wheat. Four entries (237, 1375, 1654, 1451) differ from others by rough glumes, which adhere very closely to the grain. This feature significantly hardens the process of threshing, and, on the other hand, contributes to the delay of water uptake [40, 95].

Apparently, the variability of PHS resistance of wheat-wheatgrass hybrids in our case is mainly due to diversity in the wheatgrass genetic component. We found only two alleles of wheat *TaVp-1*, namely *TaVp-1Bc* and *TaVp-1Ba*, the most common for different wheat cultivars [28, 57–59, 66, 91–94]. We did not reveal any significant differences in PHS resistance between wheat-wheatgrass entries with wheat gene alleles *TaVp-1Bc* and *TaVp-1Ba*. As relatively few wheat cultivars were used for the development of the studied wheat-wheatgrass entries, we can hardly expect a wide genetic variability of wheat genes controlling PHS in a wheat-wheatgrass hybrid genome [6, 96]. On the other hand, the probability of high polymorphism of wheatgrass genes affecting PHS in different wheat-wheatgrass hybrids is strong, because most of the partial amphiploids carry a synthetic mixed extra genome which has a different combination of chromosomes each time a partial amphiploid is produced [1–5].

Wheat-wheatgrass hybrids carry individual composition of wheatgrass chromosomes, inherited from *Th*. *intermedium* and/or *Th*. *ponticum* and derived from different subgenomes [1–5]. We may suggest that the recombination between different homeological chromosomes of wheatgrass in the genome of a wheat-wheatgrass hybrid is a rare event. Despite the PCR-based markers Vp1BB4_*Hae*III and Vivip applied in our study were developed on the basis of the *Vivparous-1* sequence, we may have estimated not only the effect of *ThVp-1* but also the effect of the linkage group 3 of *Thinopyrum* in the present study. The role of *Thinopyrum* chromosomes 3 may be similar to chromosomes 3 of wheat that harbor the majority of QTLs associated with PHS resistance [34–38, 75], transcriptional factor *TaVp-1* [28, 57–59, 66, 91–94], *TaMyb10* responsible for seed color [27] and epigenetic factors of DNA methylation *AGO802* [46]. PHS resistance can also be regulated not only by polymorphism in DNA sequence but also by epigenetic factors through DNA and histone methylation [46, 47] that have not been studied in wide hybrids yet and can have its own specificity. As PHS resistance is a complex trait regulated by multiple factors molecular makers Vp1BB4_*Hae*III and Vivip cannot be used as the only diagnostic tool in marker-assisted selection of wheat-wheatgrass hybrids or perennial wheat. However, they can be efficiently applied in breeding process after additional exploration and validation in complex with other molecular and phenotypic estimation methods to select PHS resistant wheat-wheatgrass entries and perennial wheat.

We have shown that the studied entries are diverse in wheatgrass component and that this diversity is associated with PHS resistance. The differences in PHS resistance between wheat-wheatgrass hybrids can be explained by genetic variations and/or epigenetic modifications. Genetic variations may represent variations in different levels: different combinations of wheatgrass chromosomes (the genome level), differences in the chromosomes of the homeologous group 3, where genes *Vp-1* and *R* are localized (the chromosome level) or structural and functional differences in the *Vp-1* genes (the gene level). Along with that, we have shown that the greatest resistance to PHS is innate to the entries of wheat-wheatgrass hybrids bearing haplotype *ThVp-1d* of the wheatgrass *Vp-1* gene (mean SS is 0.33, and mean GI is 0.64).

## Conclusion

We, for the first time, demonstrated the variability of wheat-wheatgrass hybrids in resistance to PHS. Most entries showed susceptibility that may pose a serious problem in wheat-wheatgrass hybrid either as a source of genetic variability in wheat breeding or as a crop in regions with conditions favorable to PHS. Interestingly, although all estimated entries have red grain associated with high seed dormancy, only few showed relatively low GI. Using PCR-based markers we demonstrated the polymorphism of the haplotypes of the wheatgrass *ThVp-1* gene in the wheat-wheatgrass hybrid collection and have revealed the significant effect of the *ThVp-1* gene on the PHS resistance. The further study may help to shed the light on the role of *ThVp-1* gene and its interaction with *TaVp-1* in regulation of seed dormancy.

## References

[1] Banks PM, Xu SJ, Wang RR, Larkin PJ. Varying chromosome composition of 56-chromosome wheat x *Thinopyrum intermedium* partial amphiploids. Genome. 1993 4;36(2):207–15. doi: 10.1139/g93-029 18469982 10.1139/g93-029

[2] Fedak G, Han F. Characterization of derivatives from wheat-*Thinopyrum* wide crosses. Cytogenet Genome Res. 2005;109(1–3):360–7. doi: 10.1159/000082420 15753597 10.1159/000082420

[3] Kroupin PY, Divashuk MG, Belov VI, Glukhova LI, Aleksandrov OS, Karlov GI. Comparative molecular cytogenetic characterization of partial wheat-wheatgrass hybrids. Russ J Genet. 2011 4;47(4):432–7. doi: 10.1134/S1022795411040077 21675238

[4] Hayes RC, Newell MT, DeHaan LR, Murphy KM, Crane S, Norton MR, et al Perennial cereal crops: An initial evaluation of wheat derivatives. Field Crops Res. 2012 7 11;133:68–89. doi: 10.1016/j.fcr.2012.03.014

[5] Larkin PJ, Newell MT, Hayes RC, Aktar J, Norton MR, Moroni SJ, et al Progress in developing perennial wheats for grain and grazing. Crop Pasture Sci. 2014 11 20;65(11):1147–64. doi: 10.1071/CP13330

[6] Tsitsin N V. Mnogoletnyaya pshenitsa. Moscow: Nauka; 1987. (In Russian).

[7] Gazza L, Galassi E, Ciccoritti R, Cacciatori P, Pogna NE. Qualitative traits of perennial wheat lines derived from different *Thinopyrum* species. Genet Resour Crop Evol. 2016 2;63(2):209–19. doi: 10.1007/s10722-015-0240-8

[8] Marquardt K, Vico G, Glynn C, Weih M, Eksvärd K, Dalin P, et al Farmer perspectives on introducing perennial cereal in Swedish farming systems: a sustainability analysis of plant traits, farm management, and ecological implications. Agroecol Sust food. 2016 5 27;40(5):432–50. doi: 10.1080/21683565.2016.1141146

[9] Lloyd S. Perennial wheat [Internet]. 2015 [cited 2017 Mar 31]. Available from: http://stud.epsilon.slu.se/7778/

[10] Adebiyi J, Olabisi LS, Snapp S. Understanding perennial wheat adoption as a transformative technology: evidence from the literature and farmers. Renew Agr Food Syst. 2016 4;31(2):101–10. doi: 10.1017/S1742170515000150

[11] Turner MK, DeHaan LR, Jin Y, Anderson JA. Wheatgrass–wheat partial amphiploids as a novel source of stem rust and Fusarium head blight resistance. Crop Sci. 2013 9;53(5):1994–2005. doi: 10.2135/cropsci2012.10.0584

[12] Kruppa K, Molnár-Láng M. Simultaneous visualization of different genomes (J, JSt and St) in a *Thinopyrum intermedium* × *Thinopyrum ponticum* synthetic hybrid (Poaceae) and in its parental species by multicolour genomic in situ hybridization (mcGISH). Comp Cytogenet. 2016 6 17;10(2):283–93. doi: 10.3897/CompCytogen.v10i2.7305 27551349 10.3897/CompCytogen.v10i2.7305 PMC4977803

[13] Li H, Wang X. *Thinopyrum ponticum* and *Th*. *intermedium*: the promising source of resistance to fungal and viral diseases of wheat. J Genet Genomics. 2009 9;36(9):557–65. doi: 10.1016/S1673-8527(08)60147-2 19782957 10.1016/S1673-8527(08)60147-2

[14] Salina EA, Adonina IG, Badaeva ED, Kroupin PY, Stasyuk AI, Leonova IN, et al A *Thinopyrum intermedium* chromosome in bread wheat cultivars as a source of genes conferring resistance to fungal diseases. Euphytica. 2015 7;204(1):91–101. doi: 10.1007/s10681-014-1344-5

[15] Lin M, Zhang D, Liu S, Zhang G, Yu J, Fritz AK, et al Genome-wide association analysis on pre-harvest sprouting resistance and grain color in U.S. winter wheat. BMC Genomics. 2016;17:794 doi: 10.1186/s12864-016-3148-6 27729004 10.1186/s12864-016-3148-6 PMC5059910

[16] Wang Y, Wang XL, Meng JY, Zhang YJ, He ZH, Yang Y. Characterization of *Tamyb10* allelic variants and development of STS marker for pre-harvest sprouting resistance in Chinese bread wheat. Mol Breed. 2016;36(11). doi: 10.1007/s11032-016-0573-9 27942244 10.1007/s11032-016-0573-9 PMC5097096

[17] Shorinola O, Bird N, Simmonds J, Berry S, Henriksson T, Jack P, et al The wheat *Phs-A1* pre-harvest sprouting resistance locus delays the rate of seed dormancy loss and maps 0.3 cM distal to the *PM19* genes in UK germplasm. J Exp Bot. 2016 7;67(14):4169–78. doi: 10.1093/jxb/erw194 27217549 10.1093/jxb/erw194 PMC5301926

[18] Gale MD, Lenton JR. Pre-harvest sprouting in wheat: a complex genetic and physiological problem affecting breadmaking quality in UK wheat. Asp Appl Biol. 1987;15:115–24.

[19] Humphreys DG, Noll J. Methods for characterization of preharvest sprouting resistance in a wheat breeding program. Euphytica. 2002 7;126(1):61–5. doi: 10.1023/A:1019671622356

[20] Imtiaz M, Ogbonnaya FC, Oman J, van Ginkel M. Characterization of quantitative trait loci controlling genetic variation for preharvest sprouting in synthetic backcross-derived wheat lines. Genetics. 2008 3;178(3):1725–36. doi: 10.1534/genetics.107.084939 18245824 10.1534/genetics.107.084939 PMC2278070

[21] Krupnov VA, Antonov GY, Druzhin AE, Krupnova OV. Preharvesting sprouting resistance of spring bread wheat carrying the 6Agi(6D) chromosome from *Agropyron intermedium*. Russ J Genet Appl Res. 2012 11;2(6):467–72. doi: 10.1134/S2079059712060093

[22] Zhang Y, Miao X, Xia X, He Z. Cloning of seed dormancy genes (*TaSdr*) associated with tolerance to pre-harvest sprouting in common wheat and development of a functional marker. Theor Appl Genet. 2014 4;127(4):855–66. doi: 10.1007/s00122-014-2262-6 24452439 10.1007/s00122-014-2262-6

[23] Flintham JE. Different genetic components control coat-imposed and embryo-imposed dormancy in wheat. Seed Sci Res. 2000 3;10(1):43–50. doi: 10.1017/S0960258500000052

[24] Warner RL, Kudrna DA, Spaeth SC, Jones SS. Dormancy in white-grain mutants of Chinese Spring wheat (*Triticum aestivum* L.). Seed Sci Res. 2000 3;10(1):51–60. doi: 10.1017/S0960258500000064

[25] Himi E, Mares DJ, Yanagisawa A, Noda K. Effect of grain colour gene (*R*) on grain dormancy and sensitivity of the embryo to abscisic acid (ABA) in wheat. J Exp Bot. 2002 7;53(374):1569–74. doi: 10.1093/jxb/erf005 12096095 10.1093/jxb/erf005

[26] Park H, Kreunen SS, Cuttriss AJ, DellaPenna D, Pogson BJ. Identification of the carotenoid isomerase provides insight into carotenoid biosynthesis, prolamellar body formation, and photomorphogenesis. Plant Cell. 2002 1 2;14(2):321–32. doi: 10.1105/tpc.010302 11884677 10.1105/tpc.010302 PMC152915

[27] Himi E, Noda K. Red grain colour gene (*R*) of wheat is a Myb-type transcription factor. Euphytica. 2005 9;143(3):239–42. doi: 10.1007/s10681-005-7854-4

[28] Yang Y, Zhao XL, Xia LQ, Chen XM, Xia XC, Yu Z, et al Development and validation of a *Viviparous-1* STS marker for pre-harvest sprouting tolerance in Chinese wheats. Theor Appl Genet. 2007 11;115(7):971–80. doi: 10.1007/s00122-007-0624-z 17712543 10.1007/s00122-007-0624-z

[29] Mohan A, Kulwal P, Singh R, Kumar V, Mir RR, Kumar J, et al Genome-wide QTL analysis for pre-harvest sprouting tolerance in bread wheat. Euphytica. 2009 8;168(3):319–29. doi: 10.1007/s10681-009-9935-2

[30] Cabral AL, Jordan MC, McCartney CA, You FM, Humphreys DG, MacLachlan R, et al Identification of candidate genes, regions and markers for pre-harvest sprouting resistance in wheat (*Triticum aestivum* L.). BMC Plant Biol. 2014 11 29;14:340 doi: 10.1186/s12870-014-0340-1 25432597 10.1186/s12870-014-0340-1 PMC4253633

[31] Cao L, Hayashi K, Tokui M, Mori M, Miura H, Onishi K. Detection of QTLs for traits associated with pre-harvest sprouting resistance in bread wheat (*Triticum aestivum* L.). Breed Sci. 2016 3;66(2):260–70. doi: 10.1270/jsbbs.66.260 27162497 10.1270/jsbbs.66.260 PMC4785003

[32] Fakthongphan J, Graybosch RA, Baenziger PS. Combining ability for tolerance to pre-harvest sprouting in common wheat (*Triticum aestivum* L.). Crop Sci. 2016 5;56(3):1025–35. doi: 10.2135/cropsci2015.08.0490

[33] Kato K, Nakamura W, Tabiki T, Miura H, Sawada S. Detection of loci controlling seed dormancy on group 4 chromosomes of wheat and comparative mapping with rice and barley genomes. Theor Appl Genet. 2001 5;102(6–7):980–5. doi: 10.1007/s001220000494

[34] Osa M, Kato K, Mori M, Shindo C, Torada A, Miura H. Mapping QTLs for seed dormancy and the *Vp1* homologue on chromosome 3A in wheat. Theor Appl Genet. 2003 5;106(8):1491–6. doi: 10.1007/s00122-003-1208-1 12750793 10.1007/s00122-003-1208-1

[35] Kulwal PL, Kumar N, Gaur A, Khurana P, Khurana JP, Tyagi AK, et al Mapping of a major QTL for pre-harvest sprouting tolerance on chromosome 3A in bread wheat. Theor Appl Genet. 2005 10;111(6):1052–9. doi: 10.1007/s00122-005-0021-4 16133317 10.1007/s00122-005-0021-4

[36] Mori M, Uchino N, Chono M, Kato K, Miura H. Mapping QTLs for grain dormancy on wheat chromosome 3A and the group 4 chromosomes, and their combined effect. Theor Appl Genet. 2005 5;110(7):1315–23. doi: 10.1007/s00122-005-1972-1 15803290 10.1007/s00122-005-1972-1

[37] Liu S, Bai G. Dissection and fine mapping of a major QTL for preharvest sprouting resistance in white wheat Rio Blanco. Theor Appl Genet. 2010 11;121(8):1395–404. doi: 10.1007/s00122-010-1396-4 20607209 10.1007/s00122-010-1396-4

[38] Zhou Y, Tang H, Cheng M-P, Dankwa KO, Chen Z-X, Li Z-Y, et al Genome-wide association study for pre-harvest sprouting resistance in a large germplasm collection of Chinese wheat landraces. Front Plant Sci. 2017;8:401 doi: 10.3389/fpls.2017.00401 28428791 10.3389/fpls.2017.00401 PMC5382224

[39] Mares D, Mrva K, Cheong J, Williams K, Watson B, Storlie E, et al A QTL located on chromosome 4A associated with dormancy in white- and red-grained wheats of diverse origin. Theor Appl Genet. 2005 11;111(7):1357–64. doi: 10.1007/s00122-005-0065-5 16133305 10.1007/s00122-005-0065-5

[40] Chen C-X, Cai S-B, Bai G-H. A major QTL controlling seed dormancy and pre-harvest sprouting resistance on chromosome 4A in a Chinese wheat landrace. Mol Breed. 2008 4;21(3):351–8. doi: 10.1007/s11032-007-9135-5

[41] Singh R, Matus-Cádiz M, Båga M, Hucl P, Chibbar RN. Identification of genomic regions associated with seed dormancy in white-grained wheat. Euphytica. 2010 8;174(3):391–408. doi: 10.1007/s10681-010-0137-8

[42] Liu S, Sehgal SK, Li J, Lin M, Trick HN, Yu J, et al Cloning and characterization of a critical regulator for preharvest sprouting in wheat. Genetics. 2013 9;195(1):263–73. doi: 10.1534/genetics.113.152330 23821595 10.1534/genetics.113.152330 PMC3761307

[43] Shorinola O, Balcárková B, Hyles J, Tibbits JFG, Hayden MJ, Holušova K, et al Haplotype analysis of the pre-harvest sprouting resistance locus Phs-A1 reveals a causal role of *TaMKK3-A* in global germplasm. Front Plant Sci. 2017 9 13;8:1555 doi: 10.3389/fpls.2017.01555 28955352 10.3389/fpls.2017.01555 PMC5602128

[44] Zheng J, Chen F, Wang Z, Cao H, Li X, Deng X, et al A novel role for histone methyltransferase KYP/SUVH4 in the control of Arabidopsis primary seed dormancy. New Phytol. 2012 2;193(3):605–16. doi: 10.1111/j.1469-8137.2011.03969.x 22122546 10.1111/j.1469-8137.2011.03969.x

[45] Liu Y, Koornneef M, Soppe WJJ. The absence of histone H2B monoubiquitination in the Arabidopsis *hub1* (*rdo4*) mutant reveals a role for chromatin remodeling in seed dormancy. Plant Cell. 2007 2;19(2):433–44. doi: 10.1105/tpc.106.049221 17329563 10.1105/tpc.106.049221 PMC1867323

[46] Singh M, Singh S, Randhawa H, Singh J. Polymorphic homoeolog of key gene of RdDM pathway, ARGONAUTE4_9 class is associated with pre-harvest sprouting in wheat (*Triticum aestivum* L.). PLoS ONE. 2013;8(10):e77009 doi: 10.1371/journal.pone.0077009 24130825 10.1371/journal.pone.0077009 PMC3793957

[47] Singh M, Singh J. Seed development-related expression of ARGONAUTE4_9 class of genes in barley: possible role in seed dormancy. Euphytica. 2012 11;188(1):123–9. doi: 10.1007/s10681-012-0624-1

[48] McCarty DR, Hattori T, Carson CB, Vasil V, Lazar M, Vasil IK. The *Viviparous-1* developmental gene of maize encodes a novel transcriptional activator. Cell. 1991 9 6;66(5):895–905. doi: 10.1016/0092-8674(91)90436-3 1889090 10.1016/0092-8674(91)90436-3

[49] Nambara E, Keith K, McCourt P, Naito S. Isolation of an internal deletion mutant of the *Arabidopsis thaliana ABI3* gene. Plant Cell Physiol. 1994 1;35(3):509–13. doi: 10.1093/oxfordjournals.pcp.a078623 8055176

[50] Jones HD, Peters NC, Holdsworth MJ. Genotype and environment interact to control dormancy and differential expression of the VIVIPAROUS 1 homologue in embryos of *Avena fatua*. Plant J. 1997 10;12(4):911–20. doi: 10.1046/j.1365-313X.1997.12040911.x 9375401 10.1046/j.1365-313x.1997.12040911.x

[51] Bailey PC, McKibbin RS, Lenton JR, Holdsworth MJ, Flintham JE, Gale MD. Genetic map locations for orthologous *Vp1* genes in wheat and rice. Theor Appl Genet. 1999 2;98(2):281–4. doi: 10.1007/s001220051069

[52] Sun Y-W, Nie L-N, Ma Y-Z, Xiu Z, Xia L-Q. Cloning and functional analysis of *Viviparous-1* promoter in wheat. Acta Agron Sin. 2011;37(10):1743–51. doi: 10.3724/SP.J.1006.2011.01743

[53] Nakamura S, Toyama T. Isolation of a *VP1* homologue from wheat and analysis of its expression in embryos of dormant and non-dormant cultivars. J Exp Bot. 2001 4;52(357):875–6. doi: 10.1093/jexbot/52.357.875 11413225 10.1093/jexbot/52.357.875

[54] McKibbin RS, Wilkinson MD, Bailey PC, Flintham JE, Andrew LM, Lazzeri PA, et al Transcripts of *Vp-1* homeologues are misspliced in modern wheat and ancestral species. Proc Natl Acad Sci USA. 2002 7 23;99(15):10203–8. doi: 10.1073/pnas.152318599 12119408 10.1073/pnas.152318599 PMC126648

[55] Xia LQ, Ganal MW, Shewry PR, He ZH, Yang Y, Röder MS. Exploiting the diversity of *viviparous-1* gene associated with pre-harvest sprouting tolerance in European wheat varieties. Euphytica. 2008 2;159(3):411–7. doi: 10.1007/s10681-007-9576-2

[56] Yang Y, Chen XM, He ZH, Röder M, Xia LQ. Distribution of *Vp-1* alleles in Chinese white-grained landraces, historical and current wheat cultivars. Cereal Res Comm. 2009;37(2):169–77. doi: 10.1556/CRC.37.2009.2.3

[57] Chang C, Feng JM, Si HQ, Yin B, Zhang HP, Ma CX. Validating a novel allele of *viviparous-1* (*Vp-1Bf*) associated with high seed dormancy of Chinese wheat landrace, Wanxianbaimaizi. Mol Breed. 2010a 3;25(3):517–25. doi: 10.1007/s11032-009-9350-3

[58] Yang Y, Zhang CL, Liu SX, Sun YQ, Meng JY, Xia LQ. Characterization of the rich haplotypes of *Viviparous-1A* in Chinese wheats and development of a novel sequence-tagged site marker for pre-harvest sprouting resistance. Mol Breed. 2014 1;33(1):75–88. doi: 10.1007/s11032-013-9935-8

[59] Divashuk M, Mayer N, Kroupin P, Rubets V, Pylnev V, Lin NTT, et al The association between the allelic state of *Vp-1B* and pre-harvest sprouting tolerance in red-seeded hexaploid triticale. Open J Genet. 2012 3 2;02(01):51 doi: 10.4236/ojgen.2012.21006

[60] Divashuk MG, Krupin PY, Fesenko IA, Belov VI, Razumova OV, Korotaeva AA, et al About possible use of Agropyron *Vp-1* (*Viviparous-1*) gens-homolog for improvement of soft wheat. Sel’skokhozyaistvennaya Biologiya [Agricultural Biology]. 2011;(5):40–4. (In Russian)

[61] Kocheshkova AA, Divashuk MG, Kroupin PYu., Karlov GI. Development of PCR-based STS marker for identification of *Viviparous-1* gene of *Thinopyrum* species in wheat background. Izv timiryazevskoi sel'skokhozyaistvennoi akademii. 2014; (5):5–12.

[62] Walker-Simmons M. Enhancement of ABA responsiveness in wheat embryos by high temperature. Plant Cell Environ. 1988 11;11(8):769–75. doi: 10.1111/j.1365-3040.1988.tb01161.x

[63] Saghai-Maroof MA, Soliman KM, Jorgensen RA, Allard RW. Ribosomal DNA spacer-length polymorphisms in barley: mendelian inheritance, chromosomal location, and population dynamics. Proc Natl Acad Sci USA. 1984 12;81(24):8014–8. 6096873 10.1073/pnas.81.24.8014 PMC392284

[64] Sturges HA. The choice of a class interval. J Am Stat Assoc. 1926 3;21(153):65–6. doi: 10.1080/01621459.1926.10502161

[65] Lee H-Y, Ro N-Y, Jeong H-J, Kwon J-K, Jo J, Ha Y, et al Genetic diversity and population structure analysis to construct a core collection from a large *Capsicum* germplasm. BMC Genetics. 2016 11 14;17:142 doi: 10.1186/s12863-016-0452-8 27842492 10.1186/s12863-016-0452-8 PMC5109817

[66] Chang C, Zhang HP, Feng JM, Yin B, Si HQ, Ma CX. Identifying alleles of *Viviparous-1B* associated with pre-harvest sprouting in micro-core collections of Chinese wheat germplasm. Mol Breed. 2010b 3 1;25(3):481–90. doi: 10.1007/s11032-009-9346-z

[67] Walker-Simmons M. ABA Levels and sensitivity in developing wheat embryos of sprouting resistant and susceptible cultivars. Plant Physiol. 1987 1 5;84(1):61–6. doi: 10.1104/pp.84.1.61 16665406 10.1104/pp.84.1.61 PMC1056528

[68] Craig F, Paulsen M. Localization and physical properties of endogenous germination inhibitors in white wheat grain. Cereal Chem. 1988;65(5):404–8.

[69] Zanetti S, Winzeler M, Keller M, Keller B, Messmer M. Genetic analysis of pre-harvest sprouting resistance in a wheat × spelt cross. Crop Sci. 2000;40(5):1406–17. doi: 10.2135/cropsci2000.4051406x

[70] King RW, Wettstein-Knowles P von. Epicuticular waxes and regulation of ear wetting and pre-harvest sprouting in barley and wheat. Euphytica. 2000 3;112(2):157–66. doi: 10.1023/A:1003832031695

[71] Kato T, Saito N, Kashimura K, Shinohara M, Kurahashi T, Taniguchi K. Germination and growth inhibitors from wheat (*Triticum aestivum* L.) husks. J Agric Food Chem. 2002 10 23;50(22):6307–12. doi: 10.1021/jf0204755 12381108 10.1021/jf0204755

[72] Bell LW, Wade LJ, Ewing MA. Perennial wheat: a review of environmental and agronomic prospects for development in Australia. Crop Pasture Sci. 2010 9 30;61(9):679–90. doi: 10.1071/CP10064

[73] Cox TS, Tassel DLV, Cox CM, DeHaan LR. Progress in breeding perennial grains. Crop Pasture Sci. 2010 7 27;61(7):513–21. doi: 10.1071/CP09201

[74] Murphy KM, Lyon SR, Balow KA, Jones SS. Post-sexual cycle regrowth and grain yield in *Thinopyrum elongatum* × *Triticum aestivum* amphiploids. Plant Breed. 2010 10;129(5):480–3. doi: 10.1111/j.1439-0523.2009.01712.x

[75] Kato K, Nakamura W, Tabiki T, Miura H, Sawada S. Detection of loci controlling seed dormancy on group 4 chromosomes of wheat and comparative mapping with rice and barley genomes. Theor Appl Genet. 2001 5;102(6–7):980–5. doi: 10.1007/s001220000494

[76] Flintham J, Adlam R, Bassoi M, Holdsworth M, Gale M. Mapping genes for resistance to sprouting damage in wheat. Euphytica. 2002 7;126(1):39–45. doi: 10.1023/A:1019632008244

[77] Groos C, Gay G, Perretant M-R, Gervais L, Bernard M, Dedryver F, et al Study of the relationship between pre-harvest sprouting and grain color by quantitative trait loci analysis in a white×red grain bread-wheat cross. Theor Appl Genet. 2002 1;104(1):39–47. doi: 10.1007/s001220200004 12579426 10.1007/s001220200004

[78] Lohwasser U, Röder MS, Börner A. QTL mapping of the domestication traits pre-harvest sprouting and dormancy in wheat (*Triticum aestivum* L.). Euphytica. 2005 9;143(3):247–9. doi: 10.1007/s10681-005-7858-0

[79] Kottearachchi NS, Uchino N, Kato K, Miura H. Increased grain dormancy in white-grained wheat by introgression of preharvest sprouting tolerance QTLs. Euphytica. 2006 12;152(3):421–8. doi: 10.1007/s10681-006-9231-3

[80] Ogbonnaya FC, Imtiaz M, Ye G, Hearnden PR, Hernandez E, Eastwood RF, et al Genetic and QTL analyses of seed dormancy and preharvest sprouting resistance in the wheat germplasm CN10955. Theor Appl Genet. 2008 5;116(7):891–902. doi: 10.1007/s00122-008-0712-8 18368385 10.1007/s00122-008-0712-8

[81] Ren X, Lan X, Liu D, Wang J, Zheng Y. Mapping QTLs for pre-harvest sprouting tolerance on chromosome 2D in a synthetic hexaploid wheat x common wheat cross. J Appl Genet. 2008;49(4):333–41. doi: 10.1007/BF03195631 19029680 10.1007/BF03195631

[82] Kumar A, Kumar J, Singh R, Garg T, Chhuneja P, Balyan HS, et al QTL analysis for grain colour and pre-harvest sprouting in bread wheat. Plant Sci. 2009 8;177(2):114–22. doi: 10.1016/j.plantsci.2009.04.004

[83] Munkvold JD, Tanaka J, Benscher D, Sorrells ME. Mapping quantitative trait loci for preharvest sprouting resistance in white wheat. Theor Appl Genet. 2009 11;119(7):1223–35. doi: 10.1007/s00122-009-1123-1 19669633 10.1007/s00122-009-1123-1

[84] Fofana B, Humphreys DG, Rasul G, Cloutier S, Brûlé-Babel A, Woods S, et al Mapping quantitative trait loci controlling pre-harvest sprouting resistance in a red × white seeded spring wheat cross. Euphytica. 2009 2;165(3):509–21. doi: 10.1007/s10681-008-9766-6

[85] Mares D, Rathjen J, Mrva K, Cheong J. Genetic and environmental control of dormancy in white-grained wheat (*Triticum aestivum* L.). Euphytica. 2009 8;168(3):311–8. doi: 10.1007/s10681-009-9927-2

[86] Chao S, Xu SS, Elias EM, Faris JD, Sorrells ME. Identification of chromosome locations of genes affecting preharvest sprouting and seed dormancy using chromosome substitution lines in tetraploid wheat (*Triticum turgidum* L.). Crop Sci. 2010 7;50(4):1180–7. doi: 10.2135/cropsci2009.10.0589

[87] Zhu Z, Tian B, Liu B, Xie Q, Tian J. Quantitative trait loci analysis for pre-harvest sprouting using intact spikes in wheat (*Triticum aestivum* L.). Shandong Agric Sci. 2010;6:19–23. Chinese

[88] Zhang HP, Feng JM, Chang C, Ma CX, Zhang XY, Yan CS, et al Investigation of main loci contributing to strong seed dormancy of Chinese wheat landrace. J Agric Biotech. 2011;19(2):270–7. Chinese

[89] Knox RE, Clarke FR, Clarke JM, Fox SL, DePauw RM, Singh AK. Enhancing the identification of genetic loci and transgressive segregants for preharvest sprouting resistance in a durum wheat population. Euphytica. 2012 7;186(1):193–206. doi: 10.1007/s10681-011-0557-0

[90] Gao X, Hu C, Li H, Yao Y, Meng M, Dong J, et al Factors affecting pre-harvest sprouting resistance in wheat (Triticum aestivum L.): a review. J Anim Plant Sci. 2013;23:556–65.

[91] Xia LQ, Yang Y, Ma YZ, Chen XM, He ZH, Röder MS, et al What can the *Viviparous-1* gene tell us about wheat pre-harvest sprouting? Euphytica. 2009 8;168(3):385–94. doi: 10.1007/s10681-009-9928-1

[92] Chang C, Zhang H-P, Zhao Q-X, Feng J-M, Si H-Q, Lu J, et al Rich allelic variations of *Viviparous-1A* and their associations with seed dormancy/pre-harvest sprouting of common wheat. Euphytica. 2011 5;179(2):343–53. doi: 10.1007/s10681-011-0348-7

[93] Wang J, Liu Y, Wang Y, Chen Z, Dai S, Cao W, et al Genetic variation of *Vp1* in Sichuan wheat accessions and its association with pre-harvest sprouting response. Genes Genom. 2011 4;33(2):139 doi: 10.1007/s13258-010-0125-3

[94] Sun YW, Jones HD, Yang Y, Dreisigacker S, Li SM, Chen XM, et al Haplotype analysis of *Viviparous-1* gene in CIMMYT elite bread wheat germplasm. Euphytica. 2012 7;186(1):25–43. doi: 10.1007/s10681-011-0482-2

[95] Kong L, Wang F, Si J, Feng B, Li S. Water-soluble phenolic compounds in the coat control germination and peroxidase reactivation in *Triticum aestivum* seeds. Plant Growth Regul. 2008 12;56(3):275 doi: 10.1007/s10725-008-9307-2

[96] Tsitsin NV, Lubimova VF. New species and forms of cereals derived from hybridization between wheat and couch grass. The American Naturalist. 1959 5;93(870):181–91.

